# Supplementary material for: The NTP generating activity of pyruvate kinase II is critical for apicoplast maintenance in Plasmodium falciparum
Source: eLife. 2020 Aug 20;9:e50807. doi: 10.7554/eLife.50807 (PMC7556864; doi:10.7554/eLife.50807)
Supplement: Table 1—source data 1. — The endonuclease sites used for cloning (EcoRI and HindIII) are shown in lower case italics and the stop codon is highlighted in red. [file elife-50807-table1-data1.pdf]

**_gaattc_GGCAGTGGAAACGGCTCAGGGGATCTGCTGTTCTTGAATAATATTAACATTAACAAAAA
TGTTGAACGCAAAAAGAATATCCTAGCCCATTCAGAAATTGGGACGGTTCGTAACATTGATGACA
TTAACCTTTTAAACCACAACAAGAAAAACGAAATTTCGTTTACGAAATGTAAACAAATCGCAACG
ATTGGTCCCGCTTCAGAAAATTTTGAACAACTTGAAAAACTGTACCTGAACGGTATTGATGTCTT
TCGTCTGAACTTTAGTCATGGCTTGAAAAGCATCAAGAAATACATTATTAATTCTATCAGGATCC
TGGAAAAAAAATATGACACCACAATCGGAATTTTAGGTGATATTCAAGGGCCGAAAATACGGATT
GGCGAATTTGAAAAAAACCAGATTAACGAAAATGATAATAATACGTTTGTTGAACTGAAAGAAGG
TGATCTGTTCAGTTTCGATCTGATGAATAGCCTGGGCAATCAGAATCGCGTGCAGCTGAATTATC
CGGAACTGATTAAGAACGCAAAAGCGGGCCAGATCATTCTGCTGGACGATGGTAACCTGAAAATG
AAAATTTTAGAAAACAACTATGATACTAGCAACATTCAGAACTCTTACATTAAAGTCCAGGTCCT
GACCGGTGGCAAACTGTACAGCAAAAAAGGCTTCTGTATCCCAAACATGATCATGCCGATTGATG
TTCTGAGCGAGAAAGATATTAAAGACATCCTGTTCTGCATCAACGAAGAAGTTGACTTCCTGGGT
TACTCCTTTGTGCAGACGGAATATGATCTGATCTTCCTGCGTAACATCATCAACGATTACTATGA
AAGCGATTTTTACAATAACATCAAACGCAAAGATCGGATTAACTTTGACCAAAACAATCTGCAGA
TGAAAACCTTTGATGACGAAAACGATTTCTATATTAAAGAAGTAAACGATTACTACAATAACTAT
TACCTGAAAAATGTTCAGAAATACAAACAGATCTATGACGTCTATAAAAATCAGGACCTTTACAT
TAATGATAATATTCAAAACCACTACAACAAAAACATCGTCACAAATATCGGTGATGATAAAAACA
TTATCGACGAACAACATATCCACAACAACAATAACAACAACATTGACAATATCATGAAAAACTAT
TCGAACCTGTATAACATTAACAAAGATCACAACAAAATTGCAATAATCTCCAAAATTGAGAAACC
GTCCGCGATCAAAAACATCGAAAATATCATCAAACTGAGTGATGGCATCATGATTGCCCGCGGTG
ATCTTGGCATAGAAACTAACCTGAGTAACCTGCCTATCCTGCAAAAAAAATTGATTAACCTCTGC
CGCATAAAATACAATAAACCGGTGATTGTGGCGACCCAGATGATGGAAAGCATGCGTTTCCTCCC
ATCGCCGACCCGTGCGGAAGTGACCGATGTGGCGACCGCCCTGTACGACGGCTCTGATTGCGTGA
TGTTATCCGCCGAAACTGCGACGGGCCAGTACCCGATTCTGACAGTATCTACCCAGAACAAAATC
ATCAAAGATGTTGAAAACGATTATTATTATTATGAGTACACCCAACGAAAAAATGATAAACTGAA
AATGTTGGATCACAAAAACAATTGCTGTCATGAGAACAAAGATATCCAACAGATCAACAAATATA
ATCATGAGTATATCCAGAACAACAAATATGAGCAGGATATCCAGAATAACATTTCTTACTTTGAT
AAACTAATCTTTAGCATTCGCGACATTAGCAATAACATTAATCTGAAAAGCATAATACTGTTCTC
TAATGAATTTAATAAAATTCAGAAACTGAGCAATCTGCGTACGAAAGCGCCGATTATTGTCATCA
CCGAGAACAAATATCTCGCCCGCAAACTGCAGCTGACCTGGGGCATTTATCCACACCTTTCCAAA
AAACAGAACCTGTTTAATCATGACTTGTTTTCGCTGATCAATTATGGCTGTGACGTGTCAAAAAA
AGAGGGTTTCGTGAACAGCCCTGATGAATATTCGTTAGTTACTTTTTCAAAAAATATTAATTCAG
CAAATCTGTTATATCTGTGTCAGCCGTGCCTCACCAACT_aagtcc_**
