# Supplementary material for: The NTP generating activity of pyruvate kinase II is critical for apicoplast maintenance in Plasmodium falciparum
Source: eLife. 2020 Aug 20;9:e50807. doi: 10.7554/eLife.50807 (PMC7556864; doi:10.7554/eLife.50807)
Supplement: Table 1—source data 2. — A maltose binding protein (MBP), along with a histidine (His) tag were appended to the N-terminus of harmonized PyrKII sequence and expressed in E. coli. The MBP-His-PyrkII fusion protein (126,273 Da) can be seen in lane 7, after purification using an MBP column. The TEV cleaved product, His-PyrKII (83,014 Da) can be seen in lane 8. Lane 10 shows the purified product and a minor band consistent with some covalent dimer (166 kDa). [file elife-50807-table1-data2.pdf]

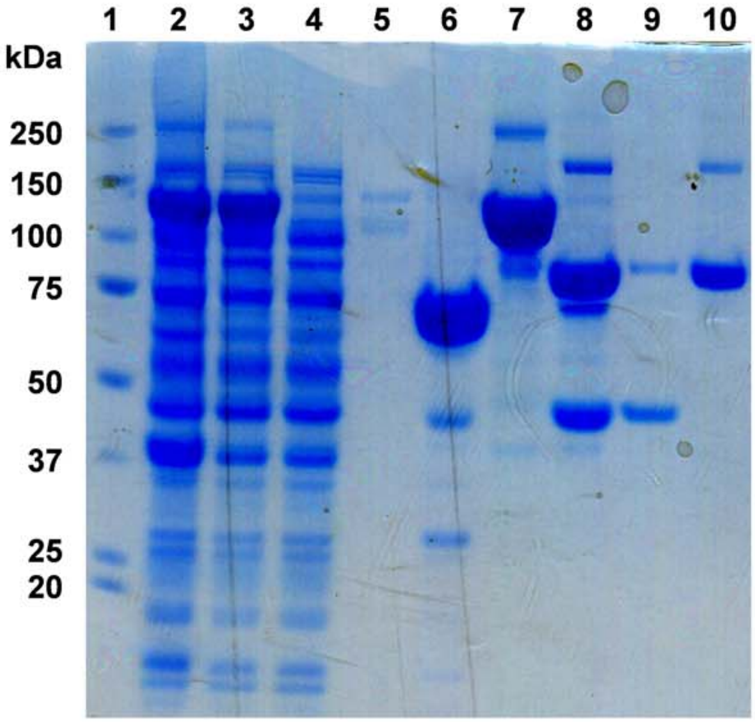

Lane	Sample
1	Ladder
2	Whole cell lysate
3	Column input (Supernatant)
4	MBP column flow through
5	MBP column wash
6	MBP-TEV
7	MBP-His-PyrKII
8	Cleaved His-PyrKII
9	Nickle column flow through
10	Purified His-PyrKII
