# Supplementary figures and images for: The NTP generating activity of pyruvate kinase II is critical for apicoplast maintenance in Plasmodium falciparum
Source: eLife. 2020 Aug 20;9:e50807. doi: 10.7554/eLife.50807 (PMC7556864; doi:10.7554/eLife.50807)

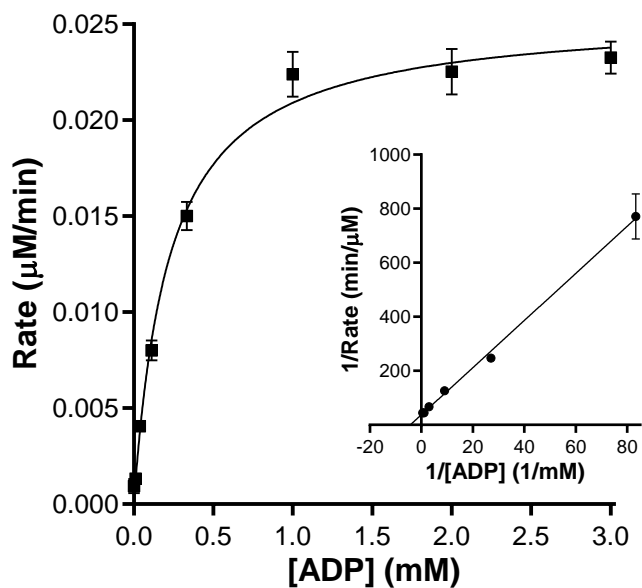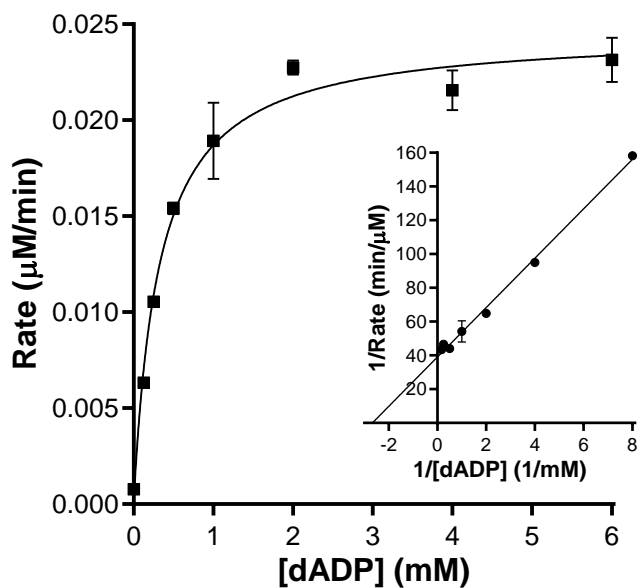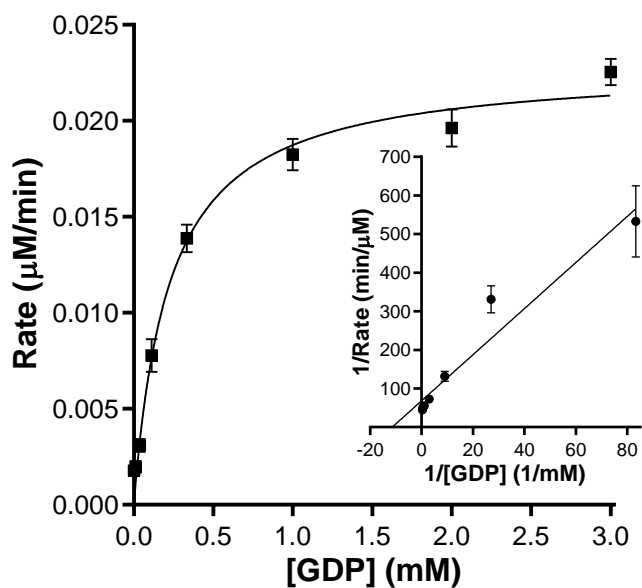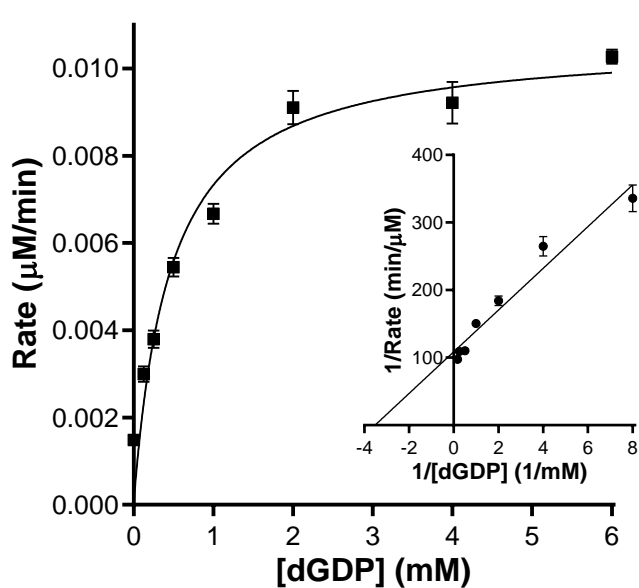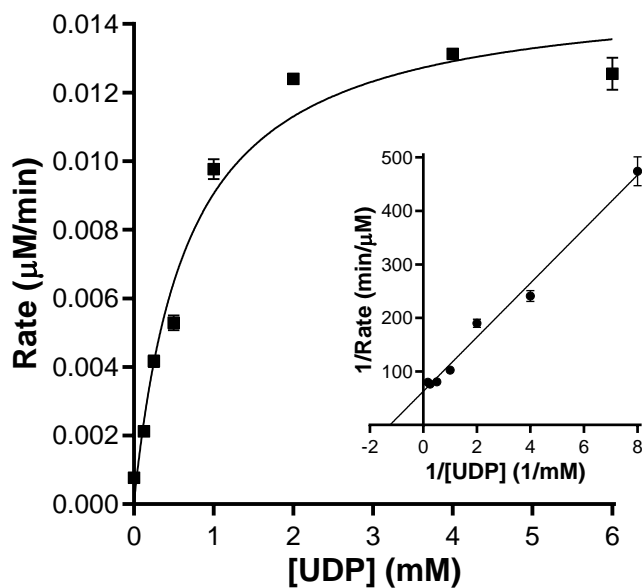

Supplement: Table 1—source data 3. — Dose-response curves from triplicate data for seven nucleotide substrates are shown fitted with hyperbolic curves based on the Michaelis-Menton equation. Insets show the same data plotted in double reciprocal (Lineweaver-Burk) plots. Error bars represent the standard error of the mean and the program Prism (GraphPad) was used to generate the plots in this figure. [file elife-50807-table1-data3.pdf]

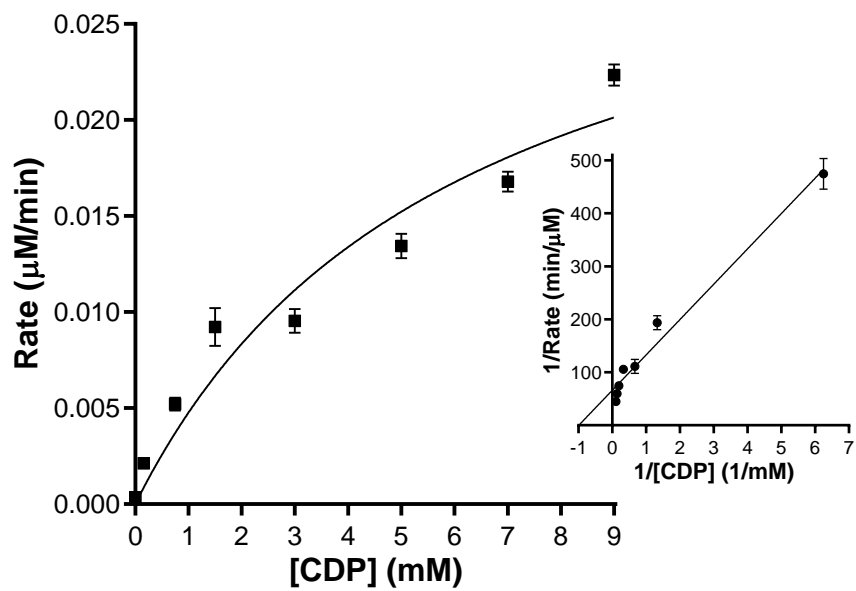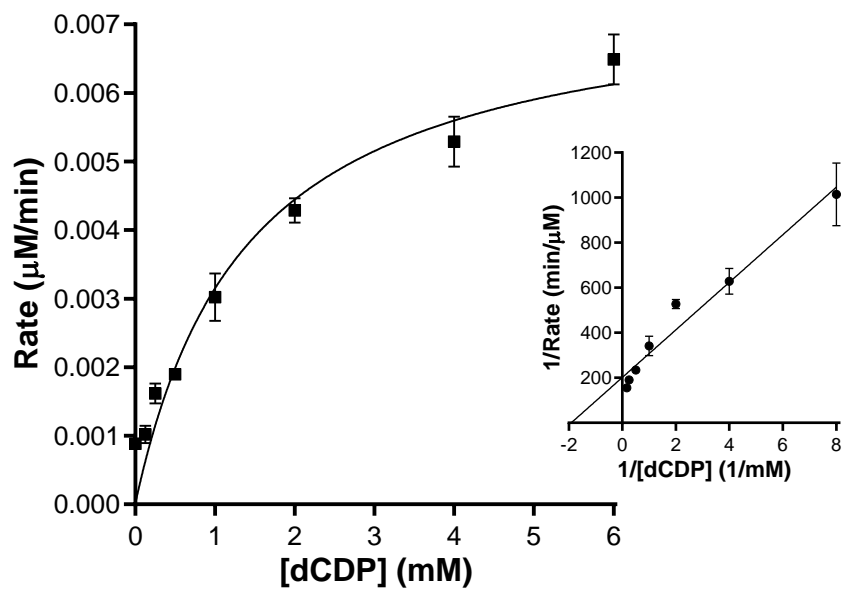

Supplement: Table 1—source data 4. — Dose-response curves from triplicate data for seven nucleotide substrates are shown fitted with hyperbolic curves based on the Michaelis-Menton equation. Insets show the same data plotted in double reciprocal (Lineweaver-Burk) plots. Error bars represent the standard error of the mean and the program Prism (GraphPad) was used to generate the plots in this figure. [file elife-50807-table1-data4.pdf]
