# Supplementary material for: The NTP generating activity of pyruvate kinase II is critical for apicoplast maintenance in Plasmodium falciparum
Source: eLife. 2020 Aug 20;9:e50807. doi: 10.7554/eLife.50807 (PMC7556864; doi:10.7554/eLife.50807)
Supplement: Transparent reporting form [file elife-50807-transrepform.docx]

***eLife’s* transparent reporting form**

We encourage authors to provide detailed information *within their submission* to facilitate the interpretation and replication of experiments. Authors can upload supporting documentation to indicate the use of appropriate reporting guidelines for health-related research (see [EQUATOR Network](http://www.equator-network.org/%20)), life science research (see the [BioSharing Information Resource](https://biosharing.org/)), or the [ARRIVE guidelines](http://www.plosbiology.org/article/info:doi/10.1371/journal.pbio.1000412) for reporting work involving animal research. Where applicable, authors should refer to any relevant reporting standards documents in this form.

If you have any questions, please consult our Journal Policies and/or contact us: [editorial@elifesciences.org](mailto:editorial@elifesciences.org).

**Sample-size estimation**

- You should state whether an appropriate sample size was computed when the study was being designed
- You should state the statistical method of sample size computation and any required assumptions
- If no explicit power analysis was used, you should describe how you decided what sample (replicate) size (number) to use

Please outline where this information can be found within the submission (e.g., sections or figure legends), or explain why this information doesn’t apply to your submission:

Power analysis was not used, as this was not a population-based study.

**Replicates**

- You should report how often each experiment was performed
- You should include a definition of biological versus technical replication
- The data obtained should be provided and sufficient information should be provided to indicate the number of independent biological and/or technical replicates
- If you encountered any outliers, you should describe how these were handled
- Criteria for exclusion/inclusion of data should be clearly stated
- High-throughput sequence data should be uploaded before submission, with a private link for reviewers provided (these are available from both GEO and ArrayExpress)

Please outline where this information can be found within the submission (e.g., sections or figure legends), or explain why this information doesn’t apply to your submission:

For the gene deletions described in Figures 2, 3, and 4 a biological replicate constitutes an independent transfection, generated at least one week apart from each other, and maintained separately from other attempts. At least two successful genetic deletion lines (biological replicates) were generated for each gene of interest.

For the growth curves in Figures 2d, 3d, 4d, 5c, and Figure 5-figure supplement 1, a biological replicate refers to a duplicated experiment separated by time, set up and run on a different day. A technical replicate refers to additional wells that were seeded on the same day and measured at the same time. For each of the curves presented, there were two biological replicates with four technical replicates each.

For the PyrKII enzyme values reported in Table 1 and figure supplements 3 and 4, technical replicates refer to enzyme from the same preparation assayed in three independent assays.

For tracking apicoplast disruption in Figure 6a and 6b, biological replicates refer to duplicated experiments separated by time, set up and run on different days. Three biological replicates were analyzed with at least 15 observations each.

For the microarray experiment in Figure 6c, biological replicates refer to duplicated experiments separated by time, with the extracted RNA processed and analyzed for each replicate on different microarray slides. The Partek Gemonomic Suite (v7.0) was used for analysis of gene expression. Within Partek, the gProcessedSignal was imported, and the intensity values were normalized to the 75th percentile, lower expressed genes were filtered out using a max cutoff of <=0.5. Data were submitted with GEO accession number GSE136169.

For the luminescence assays shown in Figure 6D, biological replicates refer to duplicated experiments separated by time, set up and run on different days. Technical replicates refer to duplicated cultures set up on the same day. Three biological replicates with three technical replicates each were analyzed.

For the RT-qPCR data shown in Figure 6-figure supplement 2, biological replicates refer to duplicated culture experiments separated by time, set up and run on different days with independent RNA extractions and cDNA synthesis. Technical replicates refer to independent qPCR experiments using these samples. Two biological replicates with three technical replicates each were analyzed.

**Statistical reporting**

- Statistical analysis methods should be described and justified
- Raw data should be presented in figures whenever informative to do so (typically when N per group is less than 10)
- For each experiment, you should identify the statistical tests used, exact values of N, definitions of center, methods of multiple test correction, and dispersion and precision measures (e.g., mean, median, SD, SEM, confidence intervals; and, for the major substantive results, a measure of effect size (e.g., Pearson's r, Cohen's d)
- Report exact p-values wherever possible alongside the summary statistics and 95% confidence intervals. These should be reported for all key questions and not only when the p-value is less than 0.05.

Please outline where this information can be found within the submission (e.g., sections or figure legends), or explain why this information doesn’t apply to your submission:

The error bars for the growth curves in Figures 2d, 3d, 4d, 5, and Figure 5-figure supplement 1 represent the standard error of the mean using the data from two biological replicates (each collected in quadruplicate). Data were analyzed by 2-way ANOVA in GraphPad Prism.

The error bars in figure 6B represent standard deviations between at least 15 observations made for each of the three biological replicates.

Standard deviations are presented for the kinetic parameters shown in Table 1. The error bars in the plots shown in Table 1-figure supplements 3 and 4 represent standard error of the mean.

For the microarray data presented in Figure 6c, a two-way ANOVA with linear contrasts for treatment (PyrKII CLD) and time (8-56hr) versus the untreated control was performed with outputs of p value, fold change, and mean ratio. Calculations are provided in a supplementary spreadsheet.

For the luminescence assays shown in Figure 6D and the RT-qPCR data shown in Figure 6-figure supplement 2, data were analyzed by 2-way ANOVA in GraphPad Prism and error bars represent the standard error of the mean.

(For large datasets, or papers with a very large number of statistical tests, you may upload a single table file with tests, Ns, etc., with reference to sections in the manuscript.)

**Group allocation**

- Indicate how samples were allocated into experimental groups (in the case of clinical studies, please specify allocation to treatment method); if randomization was used, please also state if restricted randomization was applied
- Indicate if masking was used during group allocation, data collection and/or data analysis

Please outline where this information can be found within the submission (e.g., sections or figure legends), or explain why this information doesn’t apply to your submission:

For the growth curves in Figures 2d, 3d, and 4d, parasites were grown under two different treatment conditions, one containing mevalonate supplementation, and the other containing no mevalonate supplementation.

For the growth curve in Figure 5c, and the microscopy and microarray experiments outlined in Figure 6, parasites were grown under two different treatment conditions, one containing Shield1, and the other not containing the Shield1 ligand.

For the luminescence assays shown in Figure 6D, parasites were grown under four different conditions, each of which contained anhydrotetracycline: exchange into glucose-free medium, Sheild1, fosmidomycin, and a control with no other supplements.

For the growth curve in Figure 5-figure supplement 1, parasites were grown under two different treatment conditions, one containing anhydrotetracycline, and the other not containing anhydrotetracycline.

For the RT-qPCR data shown in Figure 6-figure supplement 2, parasites were grown under two treatment conditions, one containing mevalonate, and the other without mevalonate.

Randomization of samples was not used.

**Additional data files (“source data”)**

- We encourage you to upload relevant additional data files, such as numerical data that are represented as a graph in a figure, or as a summary table
- Where provided, these should be in the most useful format, and they can be uploaded as “Source data” files linked to a main figure or table
- Include model definition files including the full list of parameters used
- Include code used for data analysis (e.g., R, MatLab)
- Avoid stating that data files are “available upon request”

Please indicate the figures or tables for which source data files have been provided:

Microarray data were submitted with GEO accession number GSE136169. Calculations are provided in a supplementary spreadsheet (Supplementary file 2).
